# Brain correlates of recall of negative autobiographical memories in patients with schizophrenia

**DOI:** 10.1192/j.eurpsy.2024.1286

**Published:** 2024-08-27

**Authors:** L. Barbosa, A. Aquino-Servín, P. Fuentes-Claramonte, M. Á. García-León, A. Karuk, N. Jaurrieta, B. Hoyas-Galán, N. Ramiro-Sousa, C. Corte-Souto, P. McKenna, E. Pomarol-Clotet

**Affiliations:** ^1^Neuroimaging, FIDMAG Sisters Hospitallers Research Foundation; ^2^Schizophrenia, CIBERSAM, ISCIII, Barcelona; ^3^Psychology, Hospital Sagrat Cor, Martorell; ^4^Nursing, Hospital General de Granollers, Granollers; ^5^Psychology, Hospital Sant Rafael; ^6^Psychology, CSMA Vila de Gràcia-Cibeles , Barcelona, Spain

## Abstract

**Introduction:**

Autobiographical memory is known to be disturbed in schizophrenia. In addition, a leading theory of auditory hallucinations (AVH) is that they are intrusive – typically negative – autobiographical memories that are misinterpreted as perceptions.

**Objectives:**

The aim of this study was to examine the brain functional correlates of recall of negatively emotionally valanced autobiographical memories in patients with schizophrenia, with a longer term aim of comparing patients with and without AVH.

**Methods:**

11 patients meeting DSM-5 criteria for schizophrenia or schizoaffective disorder and 10 age, sex and estimated premorbid IQ-matched healthy controls have so far taken part.

Participants underwent functional MRI in a 3T scanner while performing a task requiring them to recall autobiographical memories in response to individually tailored pairs of cue words. The cue words were based on autobiographical memories previously elicited in an interview with each patient and were designed to evoke the same memory. The cue words were presented in 10 20-second blocks interspersed with blocks where the subjects viewed cue words that did not evoke autobiographical memories. Brain activations were examined in three contrasts of interest: memory evoking words vs baseline, neutral words vs baseline and memory evoking vs neutral words.

Pre-processing and analysis were carried out with the FEAT module included in the FSL software. Statistical analysis was performed by means of a General Linear Model (GLM) approach.

**Results:**

In the memory evoking vs baseline contrast the patients showed hypoactivation in the medial frontal cortex compared to the healthy controls (Figure 1). There were no differences in activation between the patients and the controls comparing the memory evoking and neutral cues.

**Image:**

**Figure:**
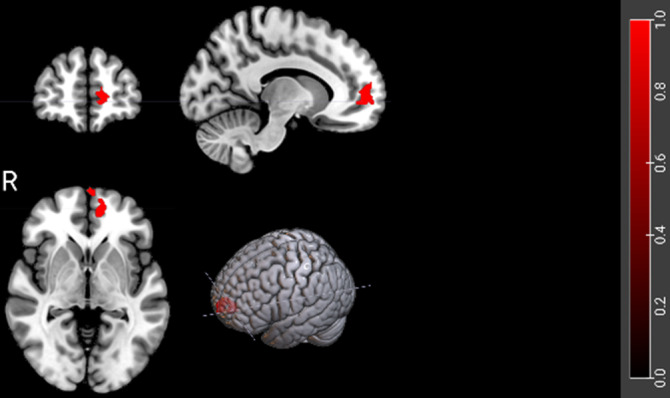

**Conclusions:**

The finding of hypoactivation in the medial frontal cortex compared to low level baseline in patients with schizophrenia suggests dysfunction in the default mode network, which is known to activate during recall of autobiographical memories.

These preliminary results suggest that recall of negative autobiographical memories in patients with schizophrenia is associated with reduced activity in the default mode network. A planned larger sample of patients and controls will be used to examine activations in patients with and without AVH.

**Disclosure of Interest:**

None Declared

